# Takayasu's arteritis presented with empyema and acute heart failure with left ventricular thrombus in a 25‐year‐old woman

**DOI:** 10.1002/kjm2.12839

**Published:** 2024-04-30

**Authors:** Yu‐Jen Chen, Chia‐Wei Hsieh, Chih‐Hung Lai, Shih‐Ting Huang

**Affiliations:** ^1^ Division of Nephrology Taichung Veterans General Hospital Taichung Taiwan; ^2^ Division of Allergy, Immunology and Rheumatology Taichung Veterans General Hospital Taichung Taiwan; ^3^ Ph.D. Program in Translational Medicine National Chung Hsing University Taichung Taiwan; ^4^ Division of Interventional Cardiology, Cardiovascular Center Taichung Veterans General Hospital Taichung Taiwan; ^5^ Institute of Clinical Medicine, National Yang‐Ming University Taipei Taiwan; ^6^ Department of Post‐Baccalaureate Medicine College of Medicine, National Chung Hsing University Taichung Taiwan

A 25‐year‐old woman presented with chronic leg edema, erythema nodosum in both lower legs for the past 6 months and recently experienced a worsening cough and numbness in her right limbs. Laboratory results indicated a serum creatinine level of 0.81 mg/dL and a urine protein/creatinine ratio of 1.8 g/g. The troponin‐T was 776.00 ng/L (<14 ng/L) and NT‐proBNP was more than 35,000 pg/mL. Chest X‐ray revealed cardiomegaly and right lower lung field consolidation, leading to her admission under the diagnosis of community‐acquired pneumonia and acute heart failure.

Further investigation through chest computed tomography (CT) uncovered right lower lung pneumonia and empyema, along with bilateral subclavian artery thrombosis. Abdominal CT revealed spleen and right kidney infarctions and diminished size and perfusion of the right kidney. Echocardiography showed chamber dilation, left ventricular (LV) generalized hypokinesis, an ejection fraction of 14.9%, and the presence of LV thrombi (Figure 1A). Blood culture yielded *Clostridium limosum*. After chest tube drainage and antibiotic treatment, the infection was controlled, and ventricular thrombi diminished with anticoagulation using warfarin targeting INR 2–2.5.

**FIGURE 1:**
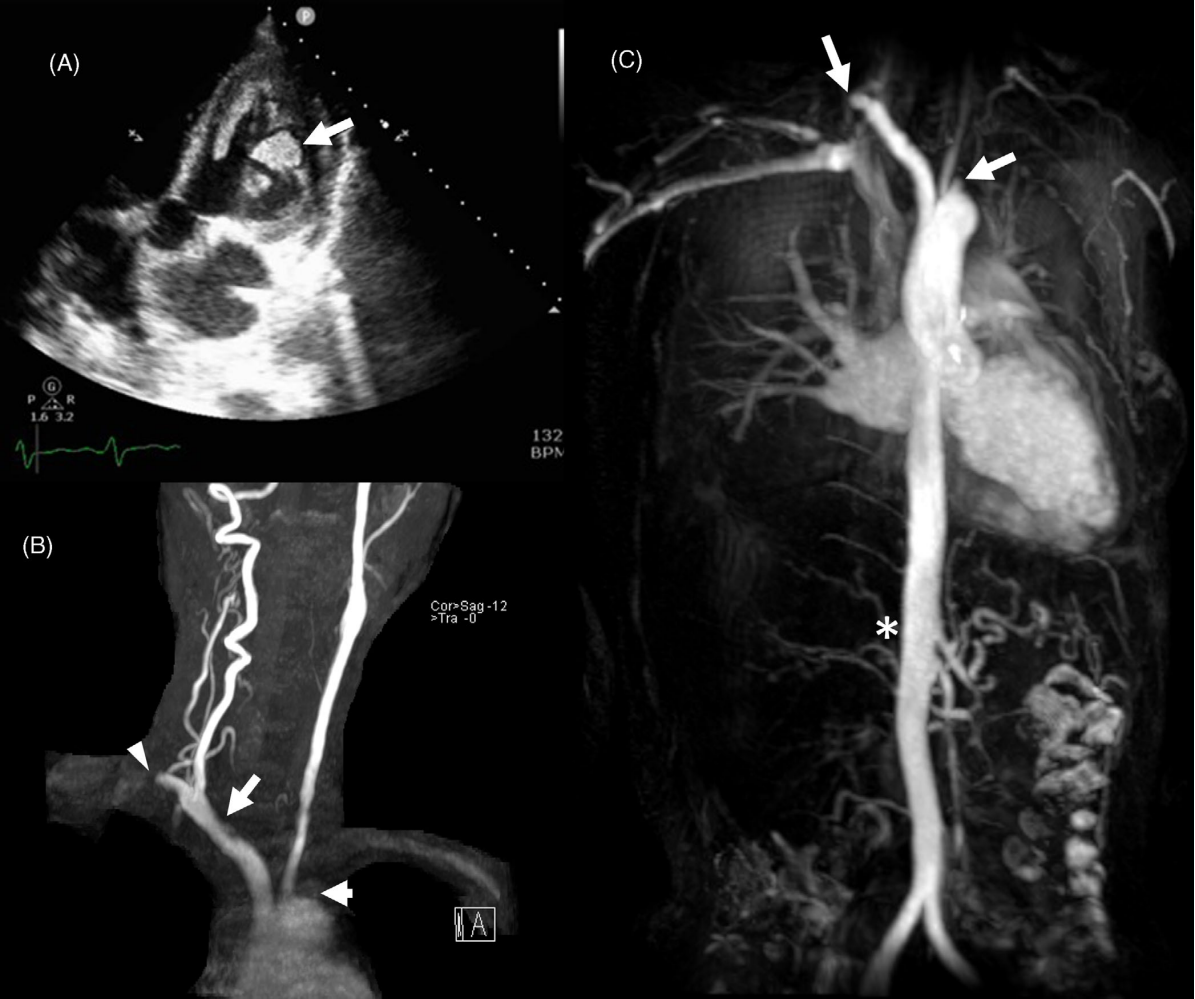
(A) Apical five chambers view of echocardiography revealed LV thrombus formation (arrow). (B) Brain MRA revealed right subclavian artery (arrowhead), right common carotid artery (long arrow), and left subclavian artery (short arrow) obstruction. (C) Thoracic MRA revealed bilateral subclavian artery obstruction(arrows), and right renal artery obstruction (asterisk). No aortic stenosis or dilation was found. LV, left ventricular; MRA, magnetic resonance angiography.

Due to multiple end‐organ damage and vascular lesions, carotid artery ultrasound and transcranial Doppler ultrasound were conducted, revealing 79% diameter stenosis and 1.7 cm intimal thickness in the left proximal common carotid artery. Arterial occlusions were observed in the left vertebral, left subclavian, right common carotid, right internal, and right external carotid arteries. Brain magnetic resonance angiography (MRA) confirmed recent infarcts, along with occlusions in the right common carotid and bilateral subclavian arteries (Figure 1B). Suspicion of large vessel arteritis led to the reevaluation of brachial artery pressure, showing pressure asymmetry. Meeting four of the six criteria according to the 1990 American College of Rheumatology classification for Takayasu arteritis,^1^ including age ≤40 years, decreased brachial artery pulse, blood pressure difference >10 mmHg between arms, and arteriogram abnormality confirmed the diagnosis of TA. Thoracic MRA displayed an absence of right renal artery flow without aorta narrowing (Figure 1C). Further arterial angiography revealed total occlusion of the right renal artery, which was unable to be intervened. Moreover, there was no coronary artery involvement which was precluded as heart failure etiology.

Upon discharge, the patient received warfarin, ivabradine, bisoprolol, and losartan. Initial immunosuppression treatment with prednisolone was gradually tapered down and combined with tocilizumab at 200 mg monthly after half year. Her ejection fraction improved to 53%, and she remains stable without signs of active disease.

Takayasu's arteritis, a systemic vasculitis with an insidious process, necessitates careful consideration by physicians. Heart failure in TA may result from various conditions, including pressure and volume overload, coronary arteritis, and myocarditis.^2^ LV thrombus formation is rare, as reported in limited studies.^3^, ^4^ In this case, the fulminant course of acute heart failure was suspected due to sepsis‐related cardiomyopathy superimposed on increased afterload. Takayasu arteritis should be considered in cases of unexplained heart failure in young women, as multiple organ damage may have occurred silently.

## CONFLICT OF INTEREST STATEMENT

The authors declare no conflicts of interest.
